# Transducin (β)-like 1 X-linked receptor 1 correlates with clinical prognosis and clinicopathological characteristics in human solid carcinomas

**DOI:** 10.18632/oncotarget.18650

**Published:** 2017-06-27

**Authors:** Fangteng Liu, Hui Gao, Yang Zhao, Zhengming Zhu

**Affiliations:** ^1^ Department of General Surgery, The Second Affiliated Hospital of Nanchang University, Nanchang 330000, Jiangxi Province, P. R. China; ^2^ The Children’s Hospital of Zhejiang University School of Medicine, Hangzhou 310052, Zhejiang Province, P. R. China; ^3^ Nanchang University School of Public Health, Nanchang 330031, Jiangxi Province, P. R. China

**Keywords:** TBL1XR1, cancer, prognosis, clinicopathology, biomarker

## Abstract

Transducin (β)-like 1 X-linked receptor 1(TBL1XR1) has been reported to be overexpressed in various human cancers, as well as contributing to carcinogenesis and progression. This synthetic analysis was performed to assess whether TBL1XR1 protein could act as a potential prognostic molecular marker for human cancers. Several online databases (PubMed, Web of Science, Embase together with Wanfang and China National Knowledge Internet database) were retrieved to identify TBL1XR1-related publications. A total of ten studies with 1837 cancer patients were included in this meta-analysis. Hazard ratios (HR) with 95% confidence intervals (CI) were applied to assess the association between TBL1XR1 expression and cancer prognosis. Odds ratios (OR) were calculated to determine the relationship between TBL1XR1 expression and clinicopathological characteristics. The overall results revealed that the overexpression of TBL1XR1 was correlated with poorer overall survival (OS) (HR: 1.77, 95% CI: 1.49–2.06, p < 0.001) and worse disease-free survival (DFS) (HR: 1.51, 95% CI: 1.19–1.84, p < 0.001) in human solid cancers. Statistical significance for OS was also found in subgroup analysis stratified by the cancer type, analysis method and follow-up time. Furthermore, elevated TBL1XR1 was associated with unfavorable clinicopathological characteristics including tumor size, depth of invasion, lymph node metastasis and TNM stage. Our meta-analysis suggested that TBL1XR1 might be served as a novel and promising biomarker to predict prognosis and clinicopathologic characteristic for cancer patients.

## INTRODUCTION

According to the recent cancer statistics figures, there were 14.1 million new cases and 8.2 million deaths in 2012 worldwide [1]. The data from the National Central Cancer Registry of China indicated that 4292,000 new cancer cases and 2814,000 cancer deaths were projected to occur in China in 2015 [2]. Although some progress has been achieved on the treatment and diagnosis, the five-year survival rate for the majority of cancer patients has remained poor. Therefore, identifying and developing novel promising prognostic biomarkers is in urgent need and of great importance for clinical practice.

Transducin β-like 1 X-linked receptor 1 (TBL1XR1), also known as transducin β-like-related protein 1(TBLR1), was an F-box/WD40-repeat containing protein [3]. Human TBL1XR1 gene was located in 3q26.32, consisting of 18 exons with the length of 178,119 bps [4]. In previous studies, TBL1XR1 was established as a core component of NCoR and SMRT repressor complexes. It has also played key roles in the regulation of several signaling pathways [5–8]. Furthermore, accumulating evidence suggested that TBL1XR1 affected tumorigenesis and cancer progression [9–10]. The dysregulated expression of TBL1XR1 was frequently observed in lesions and cancerous tissues, and it was involved in cancer development and metastasis. More and more studies reported that the expression of TBL1XR1 was associated with both clinical progression and patient survival in various human cancers [10–12]. All these results suggested that TBL1XR1 might be served as a promising and candidate prognostic marker for human cancers.

Until now, there was no consensus on the prognostic significance of TBL1XR1. Considering the limitations of discrete outcomes and small sample size in individual studies, this synthetic meta-analysis was performed to comprehensively assess the prognostic value of TBL1XR1.

## RESULTS

### Literature retrieval and analysis

**Figure 1:**
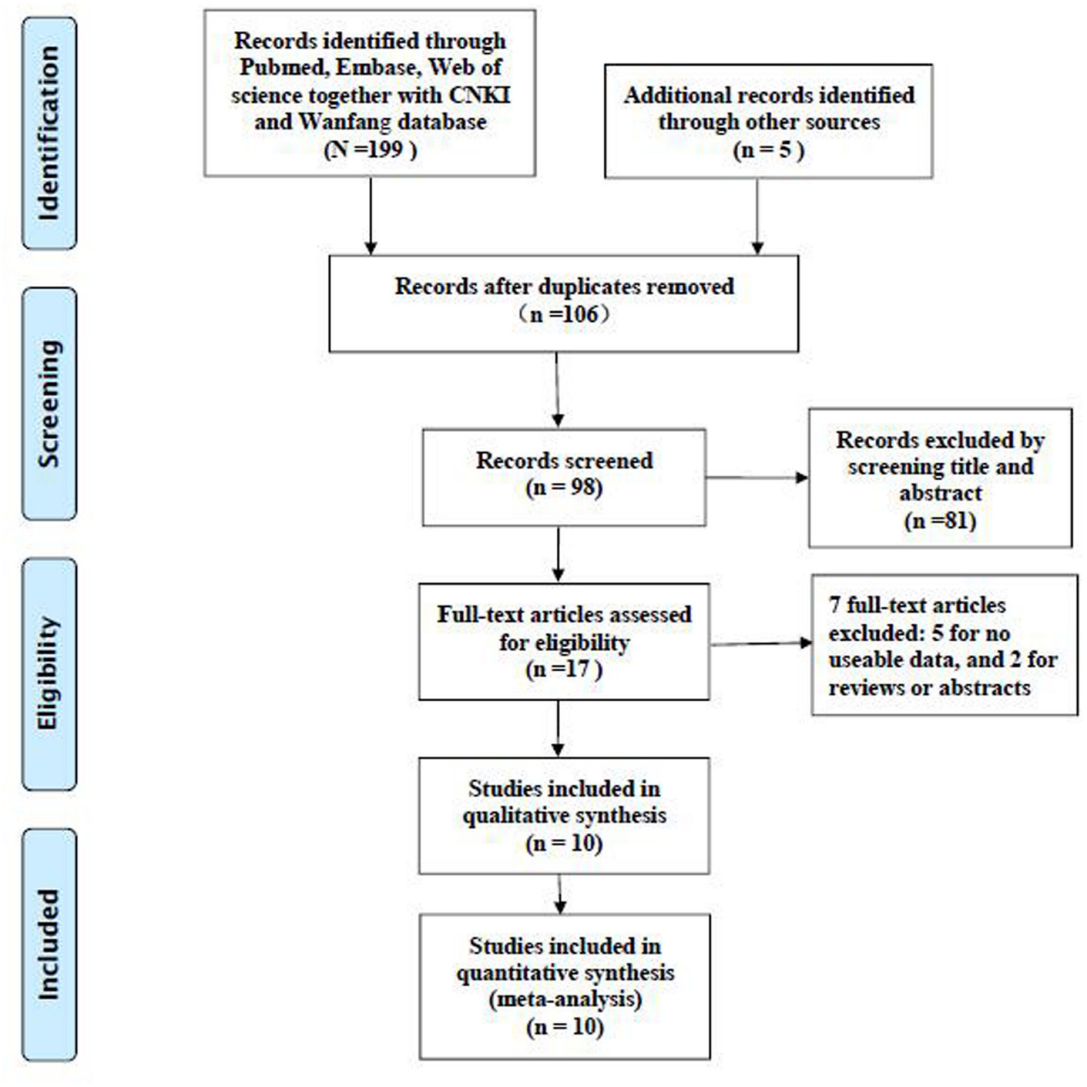
The flow diagram of the included studies

The detailed selection process has been presented (Figure 1). According to the selection criteria, ten publications written in English were finally included in this meta-analysis [10–19]. Those studies were all from P. R. China. A total of 1837 cancer patients with survival data were included in the meta-analysis. The mean sample size of patients was 183.7, with a minimum sample size of 71 and a maximum number of 334. Eight different cancer types were evaluated in our study, including esophageal squamous cell carcinoma (ESCC), gastric cancer (GC), colorectal cancer (CRC), hepatocellular carcinoma (HCC), breast cancer (BC), cervical cancer (CC), tongue squamous cell carcinoma (TSCC) and nasopharyngeal carcinoma (NPC). The publication time of those included publications was ranged from January 31^st^, 2014 to January 31^st^, 2017. The expression levels of TBL1XR1 in tissues were all determined with immunohistochemistry (IHC). The percentage of cancer patients with high TBL1XR1 expression was ranged from 33.80 % to 61.19 %. All recruited patients were pathologically diagnosed to be cancers. Main characteristics of all included studies were extracted and summarized (Table 1).

**Table 1:** Main characteristics of all the studies included in the meta-analysis

Author,Year	Country	Cancer type	Total number	Tumor stage	Follow-up (years)	High expression N (/%)	Detected method	Cut-off value	Survival analysis	Multivariate analysis
Li X, 2014	China	BC	214	I-IV	≥5 years	113(52.80%)	IHC	the average mean optical density of staining	OS	yes
Wang J, 2014	China	CC	194	Ia2-IIa2	≥5 years	108(55.67%)	IHC	NA	OS	yes
Chen SP,2014	China	NPC	106	I-IV	≥5 years	52(49.06%)	IHC	SI > 6	OS	yes
Liu L, 2015	China	ESCC	230	I-IV	≥5 years	123(53.48%)	IHC	SI ≥ 8	OS	yes
Zhou Q, 2016	China	GC	134	I-IV	<5 years	82(61.19%)	IHC	intensity score × percentage score >3	OS	no
Kuang X, 2016	China	HCC	107	A-B-C*	<5 years	52(48.60%)	IHC	the median IOD (56,299.1)	OS, DFS	OS-yes, DFS-no
Liu F, 2016	China	GC	334	I-IV	≥5 years	204(61.08%)	IHC	SI ≥8	OS	yes
Wu Y, 2016	China	TSCC	71	I-IV	<5 years	24(33.80%)	IHC	SI >6	OS, DFS	yes
Guo Y, 2016	China	HCC	310	I-IV	≥5 years	147(47.42%)	IHC	NA	OS, DFS	yes
Liu H, 2017	China	CRC	137	I-II	≥5 years	73(53.28%)	IHC	IHCS≥16	DFS	yes

BC: breast cancer; CC: cervical cancer; NPC: nasopharyngeal carcinoma; ESCC: esophageal squamous cell carcinoma; GC: gastric cancer; HCC: hepatocellular carcinoma; TSCC: tongue squamous cell carcinoma; CRC: colorectal cancer; OS: overall survival; DFS: disease-free survival; SI: staining index; IHC: immuno-histochemical staining; IOD: integrated optical density; IHCS: the final IHC score. *BCLC (Barcelona Clinic Liver Cancer).

### TBL1XR1 and clinical prognosis of human solid cancers

#### TBL1XR1 and OS

**Figure 2:**
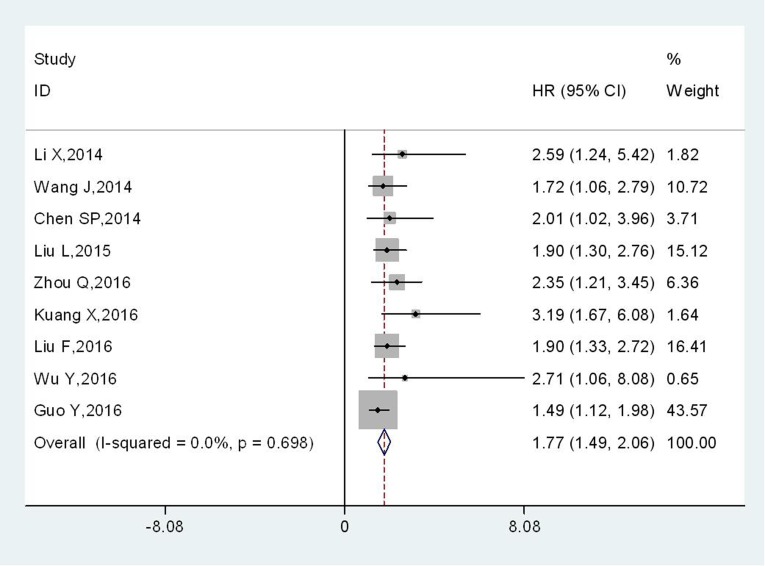
Meta-analysis of the association between TBL1XR1 and OS in various solid cancers

Nine of ten studies reported the association between TBL1XR1 and OS, with a total of 1700 cancer patients. Meta-analysis of those studies showed that high expression level of TBL1XR1 protein was associated with poorer OS in human solid cancers (HR: 1.77, 95 % CI: 1.49–2.06, p < 0.001) (Figure 2), and there was no significant heterogeneity (I^2^=0 %, P_h_=0.698; Table 2).

**Table 2:** Summary of the meta-analysis results of pooled HRs of OS of patients with elevated TBL1XR1

Stratified analysis	No. of studies	No. of patients	Pooled HR (95% CI)	p-value	Heterogeneity	
					I^2^ (%)	P_h_
[1]Cancer type						
Digestive system cancers	5	1115	1.74(1.43–2.05)	<0.001	11.9	0.338
Non-digestive system cancers	4	585	1.92(1.23–2.60)	<0.001	0.0	0.847
[2]Follow-up time						
≥ 5 years	6	1388	1.70(1.40–2.00)	<0.001	0.0	0.786
< 5 years	3	312	2.54(1.58–3.50)	<0.001	0.0	0.796
[3]Analysis type						
Multivariate	8	1566	1.73(1.44–2.03)	<0.001	0.0	0.726
Non-multivariate	1	134	1.58(1.31–3.10)	<0.001	-	-

Furthermore, subgroup analysis was performed by the cancer type. The combined HR was 1.92 (95 % CI, 1.23–2.60; p < 0.001) for non-digestive system cancers, whereas the HR was 1.74 (95 % CI, 1.43–2.05; p < 0.001) for patients with digestive system cancers (Table 2). Subgroup analysis by follow-up time revealed that the pooled HR was 1.70 (95 % CI, 1.40–2.00; p < 0.001) for studies with follow-up times ≥5 years and 2.54 (95 % CI, 1.58–3.50; p < 0.001) for studies with follow-up times <5 years (Table 2). The combined HR > 1 were observed in subgroup meta-analysis stratified by the analysis type for multivariate analysis (HR: 1.73, 95 % CI: 1.44–2.03, p < 0.001) (Table 2), suggesting that TBL1XR1 could act as an independent prognostic factor for OS (All these data were presented in Supplementary Figures 1–10).

#### TBL1XR1 and DFS

**Figure 3:**
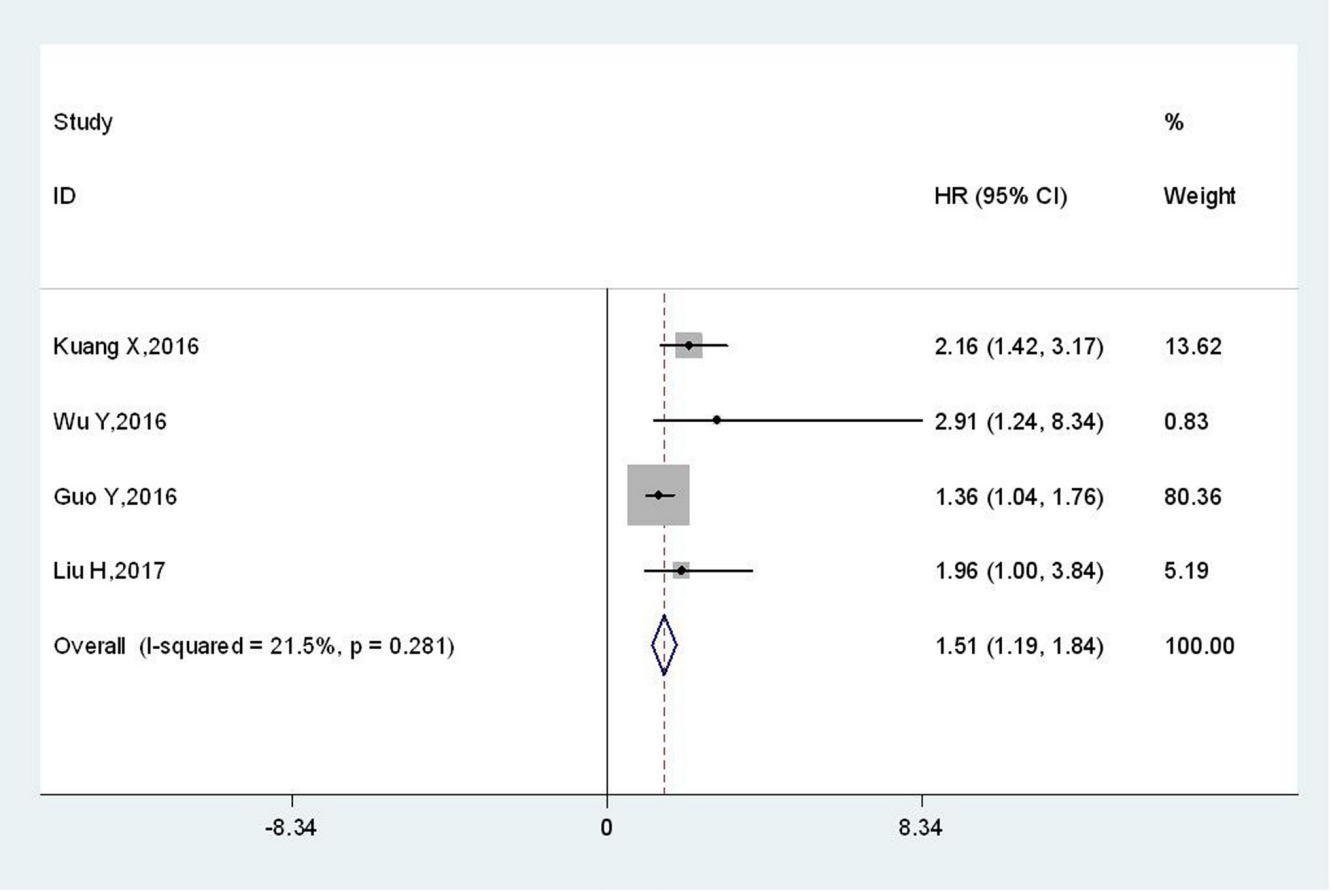
Meta-analysis of the association between TBL1XR1 and DFS

Only four studies with 625 patients reported the relationship between TBL1XR1 and DFS. Pooled results revealed that DFS was shorter in cancer patients with elevated TBL1XR1 protein (HR: 1.51, 95 % CI: 1.19–1.84, p < 0.001) (Figure 3), without significant heterogeneity among studies (I^2^=21.5 %, P_h_=0.281).

#### TBL1XR1 and clinicopathologic features of human solid cancers

The correlation of TBL1XR1 expression pattern and clinicopathological features for cancer patients was summarized (Table 3) (All these data were presented in Supplementary Figures 1–10). Our results showed that high expression of TBL1XR1 might indicate unfavorable clinicopathologic characteristics for patients with solid cancers. High expression level of TBL1XR1 was positively associated with tumor size (OR =2.56; 95 % CI, 1.51–4.35), depth of tumor (OR =2.56; 95 % CI, 1.51–4.35), lymph node metastasis (OR =3.07; 95 % CI, 1.66–5.67) and TNM stage (OR =8.48; 95 % CI, 2.25–31.97). However, elevated TBL1XR1 was not significantly correlated with age, gender or tumor differentiation.

**Table 3:** Meta-analysis of the association between TBL1XR1 and clinicopathologic features

Variable	Studies(n)	Number of patients	OR (95% CI)	p-value	Heterogeneity		
					I^2^ (%)	P_h_	Model
Age (≥60 vs. <60)	5	759	1.01(0.73-1.39)	0.96	0	0.88	Fixed effects
Gender (Male vs. Female)	8	1428	1.13(0.77-1.65)	0.54	53	0.04	Random effects
Tumor size (≥5 cm vs. <5 cm)	5	1022	2.01(1.18-3.43)	0.01	69	0.01	Random effects
Tumor invasion (T3-T4 vs.T1-T2)	5	838	2.56(1.51-4.35)	0.0005	56	0.06	Random effects
Tumor differentiation (Poorly/others vs. Well/moderately)	5	786	2.12(0.93-4.84)	0.07	85	<0.0001	Random effects
Lymph node metastasis (Yes vs. No)	6	1088	3.07(1.66-5.67)	0.0003	79	0.0002	Random effects
TNM stage (III-IV vs. I-II)	6	1088	8.48(2.25-31.97)	0.002	94	<0.00001	Random effects

#### Publication bias

**Figure 4:**
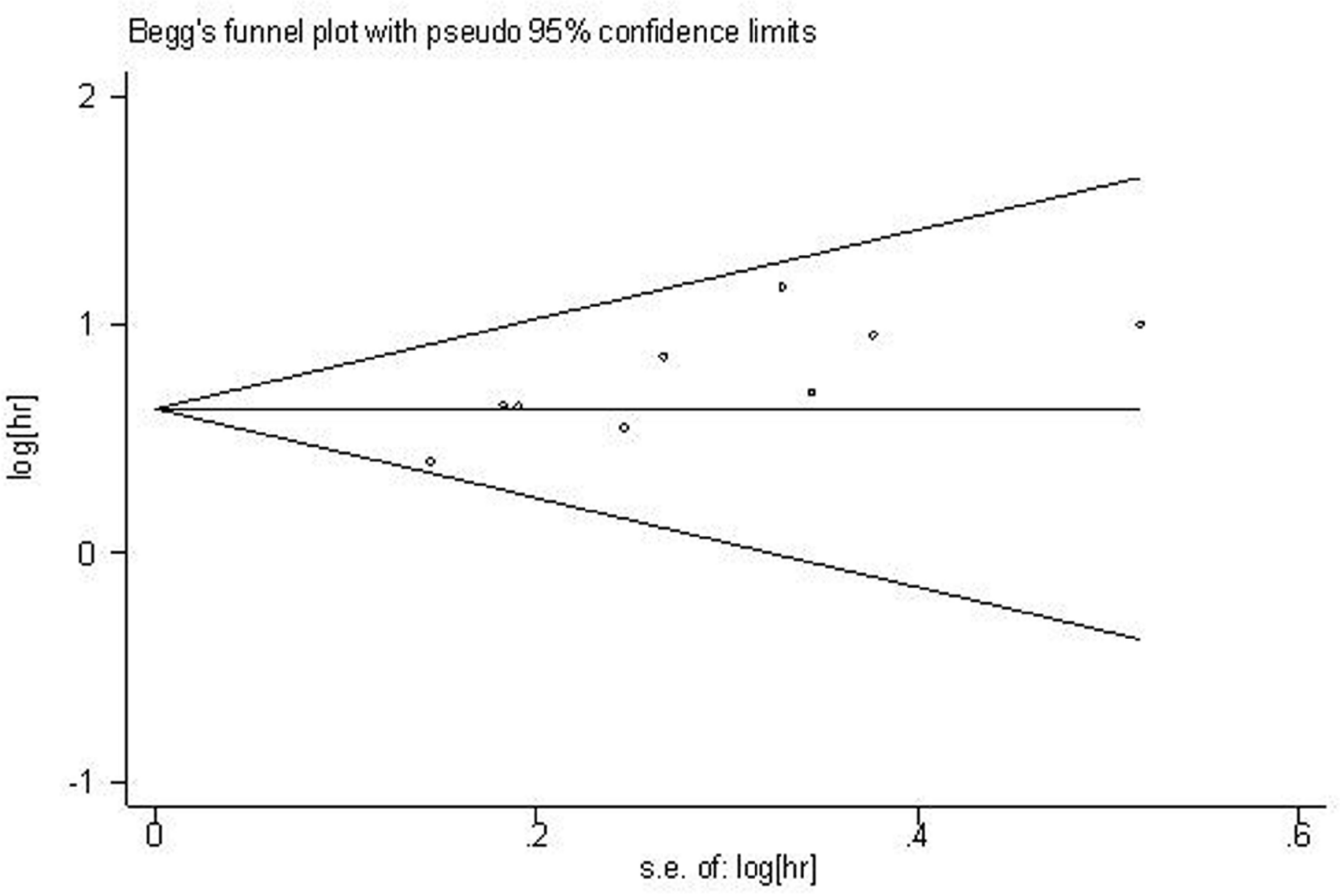
Begg’s funnel plot for the assessment of potential publication bias

**Figure 5:**
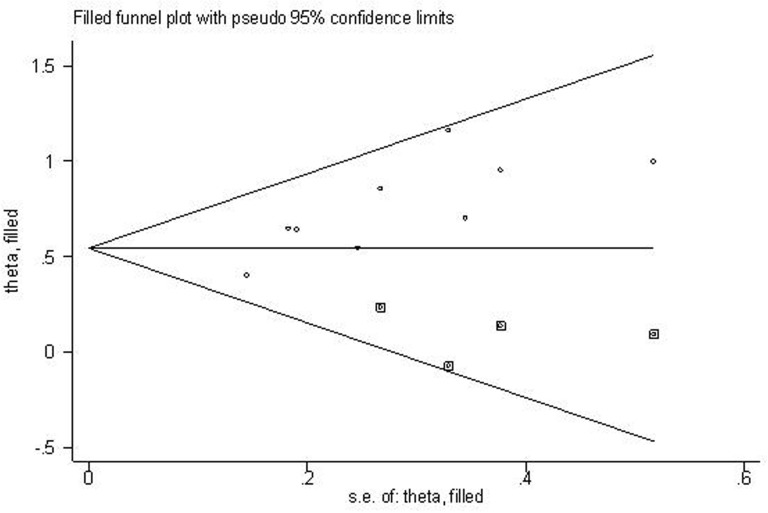
Filled funnel plot of meta-analysis with “trim-and-fill” method

Begg’s plot and Egger’s test were applied to evaluate publication bias in OS. The shape of funnel plot was asymmetrical (Figure 4), which was confirmed in Egger’s test (t(bias)=3.55, P = 0.009). Then the “trim and fill method” was also adopted to replace four missing studies (Figure 5). After correction, the adjusted pooled HR was 1.721 (95 % CI: 1.497–1.979, p < 0.001).

#### Sensitivity analysis

**Figure 6:**
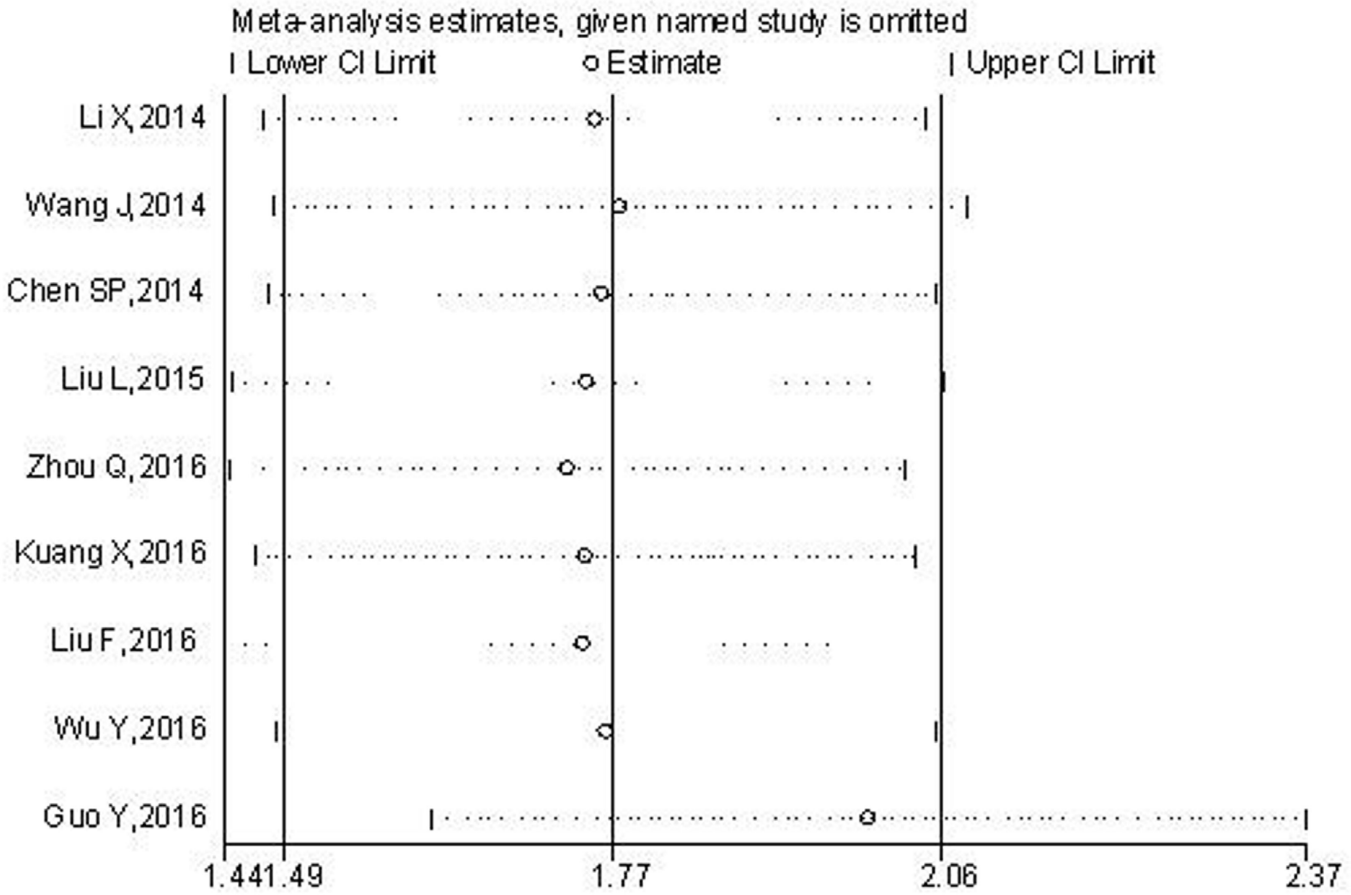
Sensitivity analysis of the relationship between TBL1XR1 expression and OS

Sensitivity analysis was conducted to assess the effect of each single study on OS. The leave-one-out sensitivity analysis showed that the pooled HRs would not be significantly varied with any individual study, suggesting the robustness of the results (Figure 6).

## DISCUSSION

Transducin-(β-) like 1 X-linked receptor 1 (TBL1XR1) was involved in multiple biological processes and played important roles in various cellular functions. TBL1XR1 could act as a transcriptional corepressor or an exchange factor of corepressors for coactivators, it could affect cell growth, anti-apoptosis, inflammation, as well as regulating the gene repression and transcription [20–23].

Recently, many studies showed that the expression of TBL1XR1 was elevated at mRNA level or protein level in multiple human cancers and the aberrant expression of TBL1XR1 could contribute to carcinogenesis and tumor progression [24–25]. As a potential predictor and therapeutic target in cancers, TBL1XR1 protein has attracted growing attentions. Several studies investigated the association between TBL1XR1 protein expression and clinical outcomes in various cancers. Chen et al*.* [12] reported that TBL1XR1 protein expression was significantly correlated with T classification, N classification, TNM stage and patient survival in NPC patients. Liu et al*.* [16] also observed the clinical relevance of TBL1XR1 expression in GC progression and found the high TBL1XR1 expression was related with positive lymph node metastasis, advanced clinical stage and inferior overall survival, while it was not associated with gender, age, tumor size and histological type. However, the study from Kuang [15] showed that there was an association between TBL1XR1 expression and tumor size, histological grade, disease stage and prognosis in HCC. In human breast cancer, Li et al*.* [10] indicated that the expression level of TBL1XR1 protein was markedly related with pathological differentiation, cerbB2 expression as well as tumor stage and prognosis. TBL1XR1 has been generally considered as predictor for prognosis, however, the case was different according to cancer types. The effects of TBL1XR1 in prostate cancer (PC) have been noteworthy, nuclear TBLR1 acted as a tumor suppressor in PC. TBLR1 physically interacts with androgen receptor, directly and selectively activating androgen-regulated genes important for growth suppression/differentiation [21–23]. With all these result, it can be seen that the clinical significance of TBL1XR1 was inconclusive or even contradictory, more dedicated-designed studies and trials should be performed before the application of TBL1XR1 as prognosis predictor for a certain cancer type. The role of TBL1XR1 as a potential prognostic marker was needed to be further studied.

As far as we know, this is the first meta-analysis to integrally assess the prognostic and clinicopathological significance of TBL1XR1 in human solid cancers. Ten studies with a total of 1837 cases were included in this meta-analysis. For the correlations between TBL1XR1 expression and prognosis, it observed that when compared with those with low expression, poorer OS (HR: 1.77, 95 % CI: 1.49–2.06, p < 0.001) and shorter DFS (HR: 1.51, 95 % CI: 1.19–1.84, p < 0.001) were observed in patients with high TBL1XR1 expression level, compared to those with low expression. According to the subgroup analysis for OS, TBL1XR1 might be served as a promising biomarker for GC (HR: 1.74, 95 % CI: 1.43–2.05; p < 0.001) and acted as an independent prognostic factor (HR: 1.73, 95 % CI: 1.44–2.03, p < 0.001). Meanwhile, high TBL1XR1 expression level also indicated a shorter five-year OS for cancer patients (HR: 1.70, 95 % CI: 1.40–2.00; p < 0.001). Furthermore, the clinicopathological value of TBL1XR1 was confirmed in tumor size, depth of invasion, lymph node metastasis and TNM stage, suggesting that TBL1XR1 was involved in tumor progression and its overexpression was associated with unfavorable clinicopathologic features.

Although the precise functions of TBL1XR1 in tumorigenesis and progression have not been clarified, a number of researches have investigated the potential roles of TBL1XR1 in tumorigenesis, invasion and metastasis. TBL1XR1 is positively correlated with aggressive behavior of tumors and its overexpression might be a crucial factor in the development of various human cancers. Many studies tried to explore the potential mechanism from molecular pathways. Several cancer-related signaling pathways have been reported to be activated by TBL1XR1. Liu et al*.* [13] found that TBL1XR1 was upregulated in ESCC cells and tissues. The elevated TBL1XR1 could promote lymphangiogenesis and lymphatic metastasis in ESCC via upregulation of VEGF-C, which had close relations with cancer metastasis and prognosis [26–28]. Liu et al*.* [19] demonstrated that TBL1XR1 could mediate the process of lymph node metastasis in CRC by regulating the expression of VEGF-C and epithelial-mesenchymal transition proteins. A newly-published study indicated that TBL1XR1 was positively associated with the lymph node metastasis of serous epithelial ovarian cancer (EOC). The result was further confirmed in human EOC cell lines. VEGF-C expression could be decreased after the silencing of TBL1XR1, suggesting that TBL1XR1 may function as an upstream regulator of VEGF-C in EOC. Furthermore, the proliferation and invasion of EOC cells were inhibited by TBL1XR1 silencing [29].

Guo et al*.* [18] also confirmed the oncogenic functions of TBLR1 in HCC and reported that the capacities of proliferation, anti-apoptosis and angiogenesis of HCC cells were significantly suppressed by silencing TBLR1 gene, which was related to the regulation of the Wnt/b-catenin pathway. Kuang [15] further verified that the activation of Wnt/b-catenin pathway was essential for TBLR1-induced EMT in HCC. And in human cervical cancer, TBLR1 protein was supposed to be a vital role in the invasion and metastasis of and it could promote epithelial–mesenchymal transition which via the NF-kB and Wnt/b-Catenin signaling pathway [11]. Similarly, TBLR1 was implicated in the development and progression of breast cancer via the activation of the β-catenin signaling pathway and cyclin D1-transactivation [10]. Additionally, ERK1/2 pathway and NF-κB pathway have been reported to be activated by TBL1XR1 in GC and NPC, suggesting that TBL1XR1 played an important role in cancer progression and drug resistance and may act as a new therapeutic target for those cancers [12, 14].

It should be noted that there were still several limitations in present meta-analysis. Firstly, although a number of major databases have been retrieved, the total sample size summarized here was relatively small. Secondly, all included patients were Chinese thus limiting the applicability of our findings to other populations. Thirdly, the cut-off value of TBL1XR1 protein expression varied in studies since standards were hard to be unified. Fourthly, a potential publication bias might exist and this was explained with trim and fill method, while the robustness of overall results has been confirmed by the sensitivity analysis.

In conclusion, from the available evidence, we found that high TBL1XR1 expression level was associated with poor clinical outcomes in cancer patients, and TBL1XR1 might be served as a valuable tumor biomarker for prognosis and clinicopathology. Certainly, well-designed studies with larger sample and other ethnic groups would be necessary to further confirm its prognostic value in different kinds of cancers.

## MATERIALS AND METHODS

### Literature retrieval to identify relevant studies for meta-analysis

An integrated retrieval was performed in three major world-wide databases (PubMed, Embase and Web of Science) and two main Chinese databases (Wanfang and CNKI). The combination of the following search terms was applied in literature retrieval: “transducin (β)-like 1 X-linked receptor 1”, “transducin beta-like 1X-linked receptor 1”, “transducin b-like protein 1-related protein”, “TBLR1” or “TBL1XR1”; and “prognosis”, “prognostic”, “survival” or “clinical outcome”; “tumor”, “neoplasm”, “cancer” or “carcinoma”. The reference lists of relevant articles were also hand-searched. The published language was limited to English and Chinese. The cutoff date for the search was January 31^st^, 2017.

### Selection criteria for study inclusion

Studies were selected for eligibility according to the following criteria:(1) Studies exploring the association between TBL1XR1 protein and human solid cancers; (2) Patients were divided into two groups (high/low) according to the expression of TBL1XR1 protein in tissue specimens; (3) Correlation of TBL1XR1 protein with overall survival (OS) or disease-free survival (DFS) was studied; (4) Sufficient data were reported for calculating the hazard ratio (HR) with 95 % CI for survival rates. The articles were excluded for reviews, case reports and conference abstracts, or without usable data for calculating hazard ratio (HR) with 95 % CI.

### Data extraction and quality assessment

The following information was extracted by two investigators (LFT and GH) independently: authors, year of publication, country, cancer type, sample size, the expression pattern of TBL1XR1, detection method, cut-off definition, follow-up time and survival data. Additionally, the clinicopathological features (such as gender, age, tumor size, infiltration depth, histopathological grade, lymph node metastasis and TNM stage) were also obtained from all included studies.

The HRs associated with upregulated TBL1XR1 expression for OS or DFS along with their 95 % CIs were directly obtained from studies with a multivariate regression analysis, otherwise, data were extracted from Kaplan-Meier curves to extrapolate HRs with 95 % CIs.

To ensure study quality, the Newcastle-Ottawa quality assessment scale (NOS) was applied to evaluate the included studies. A study with NOS scores great than or equal to 6 were considered as high-quality research. In this meta-analysis, the score of all included studies was varied from 6 to 9, with a mean value of 7.5.

### Statistical analysis

Stata statistical software version 12.0 (Stata Corporation, College Station, Texas, USA) was applied to assess the association between TBL1XR1 and OS/DFS in human solid cancers. RevMan5.3 software (Cochrane Collaboratoin) was applied to explore the relation between TBL1XR1 protein expression and clinicopathological features.

The heterogeneity across-studies was identified via I^2^ statistics and chi-square Q test. If significant heterogeneity was observed between-studies (P_h_ <0.05 or I^2^ >50 %), a random-effects model was utilized; otherwise, the fixed-effects model was applied.

Subgroup analysis was performed to further examine the prognostic value of TBL1XR1. Sensitivity analysis was applied to evaluate the stability of the results. The publication bias was assessed with Begg’s funnel plot and Egger’s test. All p values were calculated with a two-sided test. The p-value < 0.05 was considered to be statistically significant.

## SUPPLEMENTARY MATERIALS FIGURES


