# Supplementary material for: Transducin (β)-like 1 X-linked receptor 1 correlates with clinical prognosis and clinicopathological characteristics in human solid carcinomas
Source: Oncotarget. 2017 Jun 27;8(37):61626–36. doi: 10.18632/oncotarget.18650 (PMC5617451; doi:10.18632/oncotarget.18650)
Supplement: Supplementary file 1 [file oncotarget-08-61626-s001.pdf]

Transducin (β)-like 1 X-linked receptor 1 correlates with clinical prognosis and clinicopathological characteristics in human solid carcinomas

SUPPLEMENTARY MATERIALS

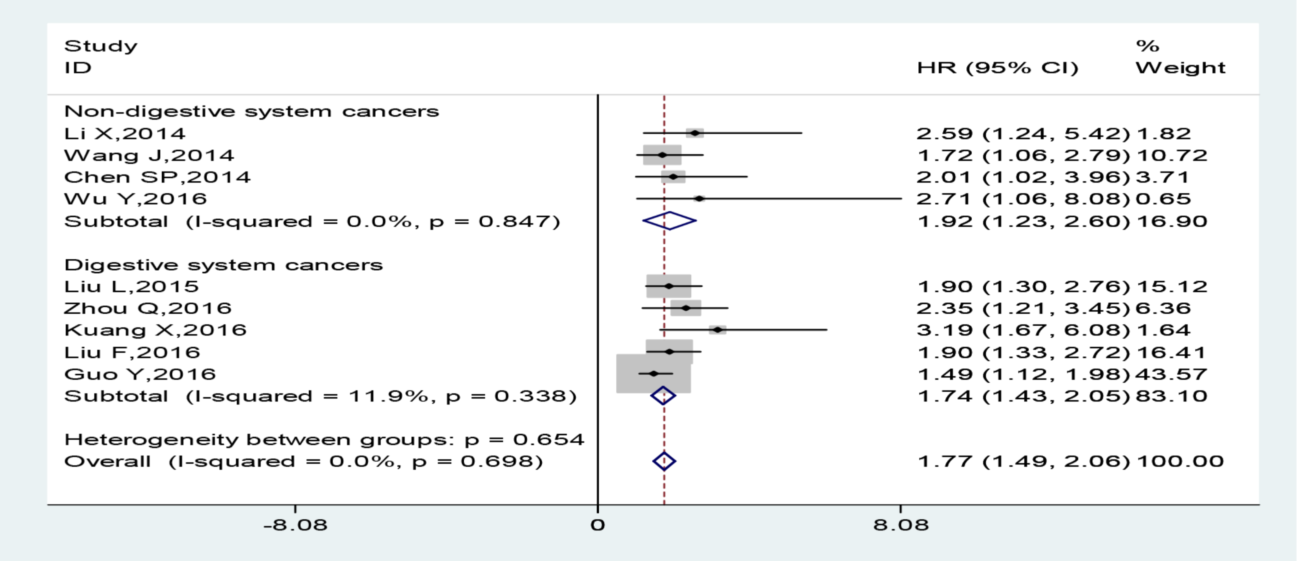

Supplementary Figure 1: Meta-analysis for the pooled HRs of OS stratified by the cancer type.

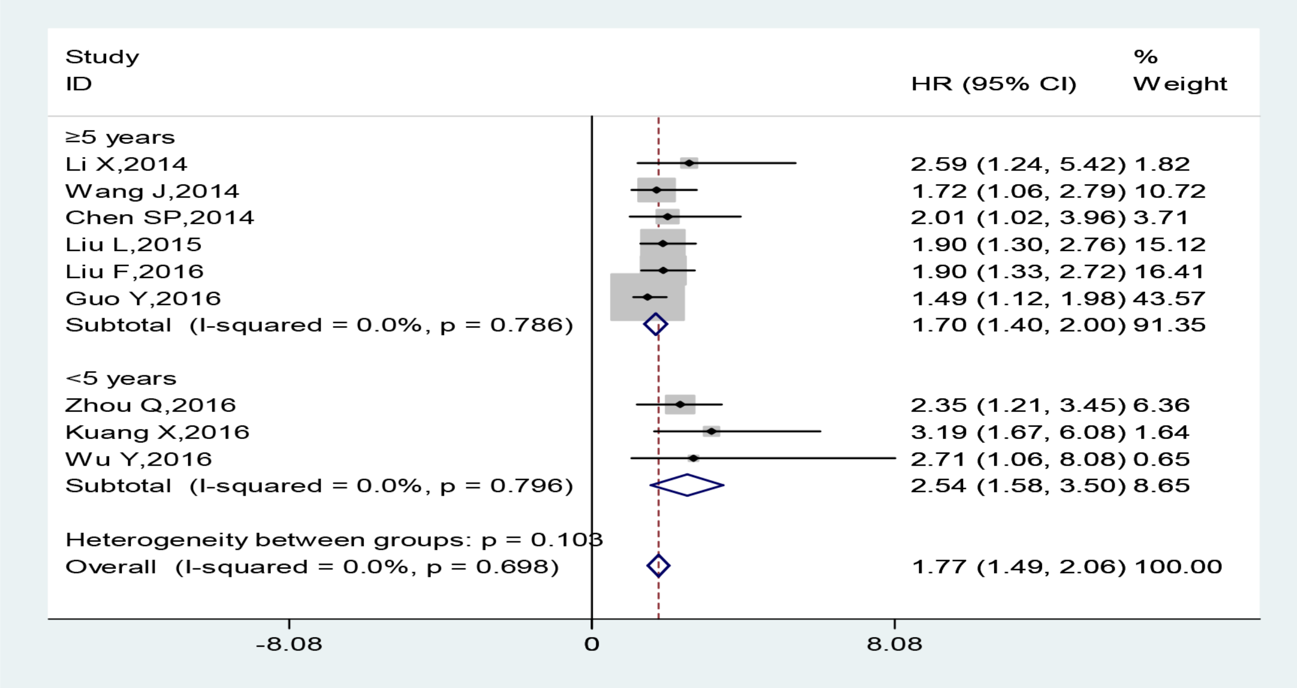

Supplementary Figure 2: Meta-analysis for the pooled HRs of OS stratified by the follow-up time.

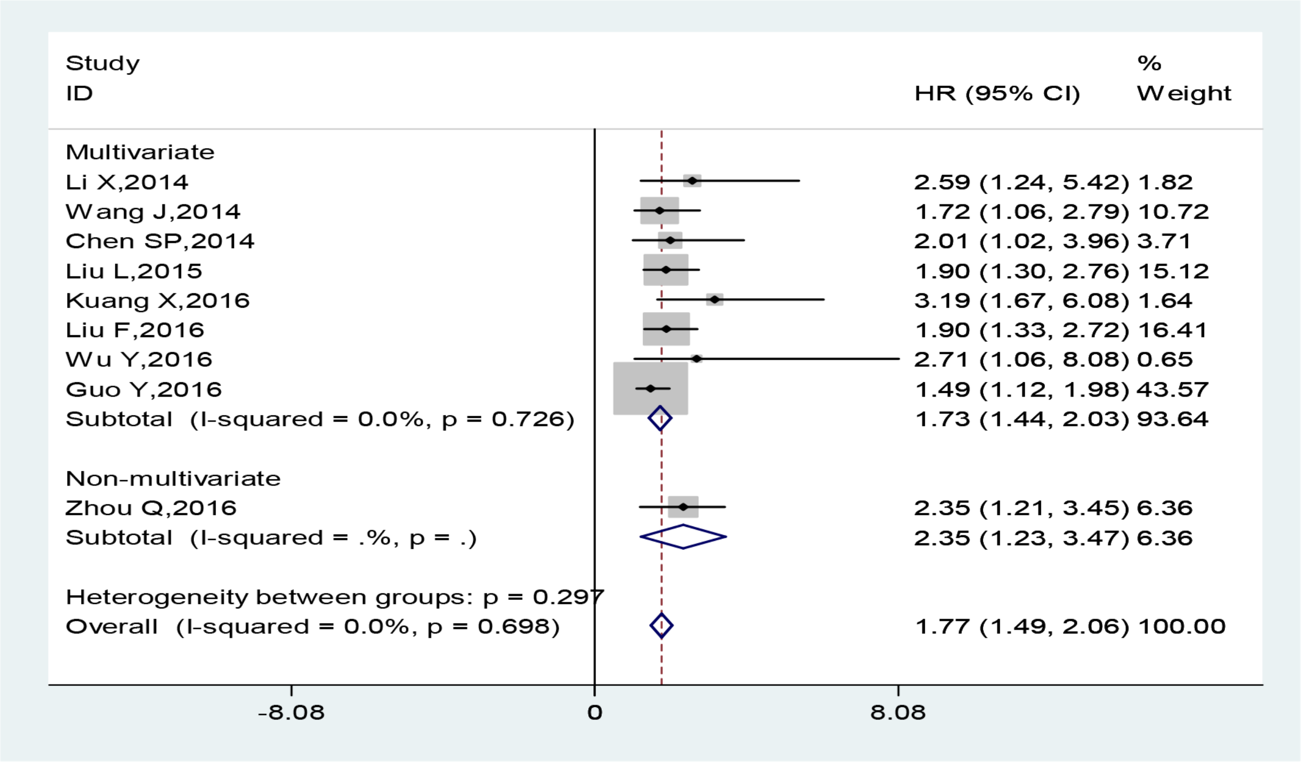

Supplementary Figure 3: Meta-analysis for the pooled HRs of OS stratified by the analysis type.

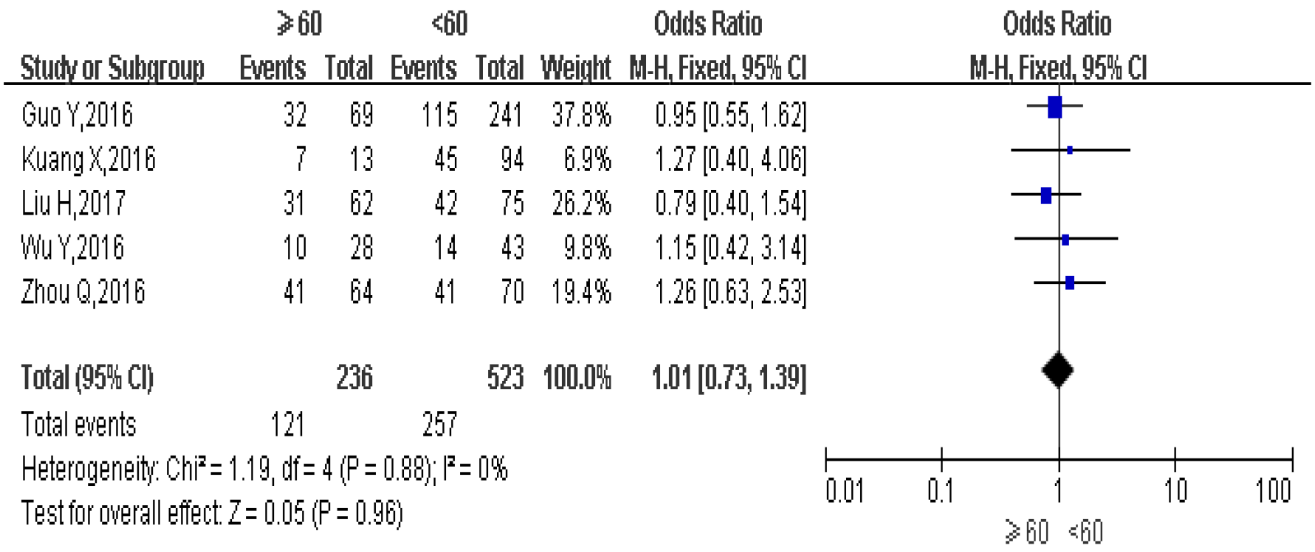

Supplementary Figure 4: The correlation between TBL1XR1 expression and age.

	Male		Female			Odds Ratio
Study or Subgroup	Events	Total	Events	Total	Weight	M-H, Random, 95% CI
Chen SP,2014	38	79	14	26	10.6%	0.79 [0.33, 1.93]
Guo Y,2016	133	273	14	37	13.5%	1.56 [0.77, 3.16]
Kuang X,2016	47	93	5	14	7.5%	1.84 [0.57, 5.90]
Liu F,2016	134	233	70	101	17.5%	0.60 [0.36, 0.98]
Liu H,2017	46	90	27	47	13.4%	0.77 [0.38, 1.58]
Liu L,2015	95	168	28	62	15.7%	1.58 [0.88, 2.84]
Wu Y,2016	13	41	11	30	9.3%	0.80 [0.30, 2.16]
Zhou Q,2016	64	95	18	39	12.5%	2.41 [1.12, 5.16]
**Total (95% CI)**		**1072**		**356**	**100.0%**	**1.13 [0.77, 1.65]**
Total events	570		187			

Heterogeneity: $Tau^2 = 0.15$; $Chi^2 = 14.78$, df = 7 (P = 0.04); $I^2 = 53\%$
Test for overall effect: Z = 0.61 (P = 0.54)

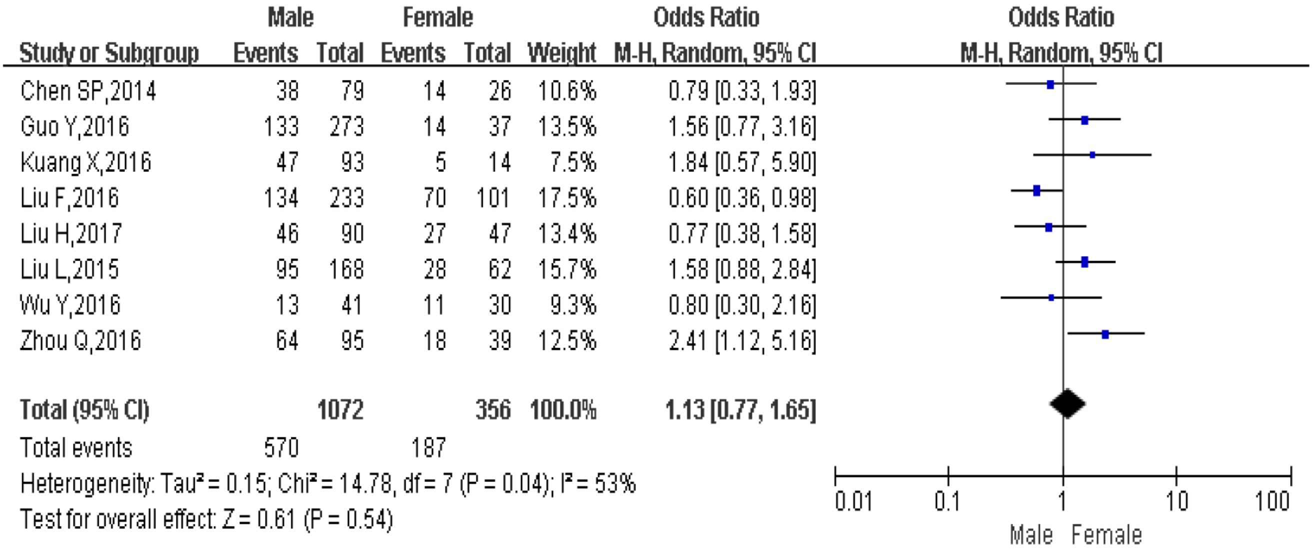

Supplementary Figure 5: The correlation between TBL1XR1 expression and gender.

	≥5 cm		<5 cm			Odds Ratio
Study or Subgroup	Events	Total	Events	Total	Weight	M-H, Random, 95% CI
Guo Y,2016	119	236	28	74	23.5%	1.67 [0.98, 2.85]
Kuang X,2016	49	83	3	24	11.0%	10.09 [2.79, 36.52]
Liu F,2016	102	149	102	185	25.2%	1.77 [1.12, 2.77]
Liu H,2017	46	70	27	67	20.2%	2.84 [1.42, 5.68]
Zhou Q,2016	41	69	41	65	20.2%	0.86 [0.43, 1.72]
**Total (95% CI)**		**607**		**415**	**100.0%**	**2.01 [1.18, 3.43]**
Total events	357		201			

Heterogeneity: Tau² = 0.24; Chi² = 13.05, df = 4 (P = 0.01); I² = 69%
Test for overall effect: Z = 2.56 (P = 0.01)

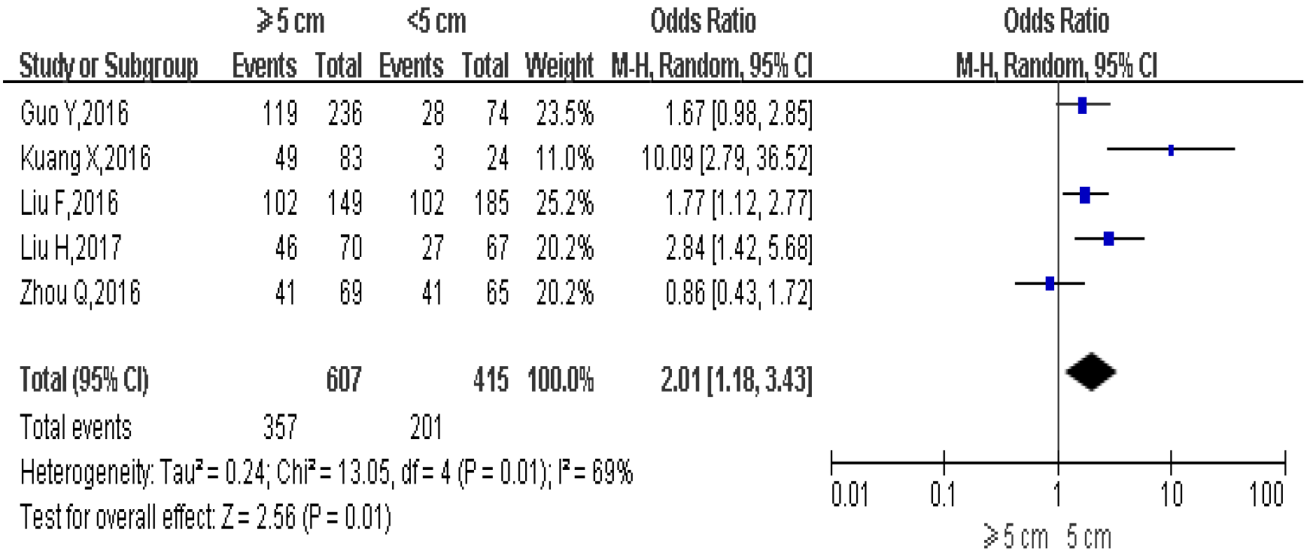

Supplementary Figure 6: The correlation between TBL1XR1 expression and tumor size.

	T3/T4		T1/T2			Odds Ratio	
Study or Subgroup	Events	Total	Events	Total	Weight	M-H, Random, 95% CI	Year
Li X,2014	29	32	84	182	12.4%	11.28 [3.32, 38.35]	2014
Chen SP,2014	41	70	11	35	18.8%	3.08 [1.31, 7.27]	2014
Liu L,2015	89	164	34	84	27.0%	1.75 [1.02, 2.97]	2015
Zhou Q,2016	67	101	15	33	20.1%	2.36 [1.06, 5.26]	2016
Liu H,2017	27	44	46	93	21.7%	1.62 [0.78, 3.37]	2017
**Total (95% CI)**		**411**		**427**	**100.0%**	**2.56 [1.51, 4.35]**	
Total events	253		190				

Heterogeneity: Tau² = 0.20; Chi² = 9.04, df = 4 (P = 0.06); I² = 56%

Test for overall effect: Z = 3.49 (P = 0.0005)

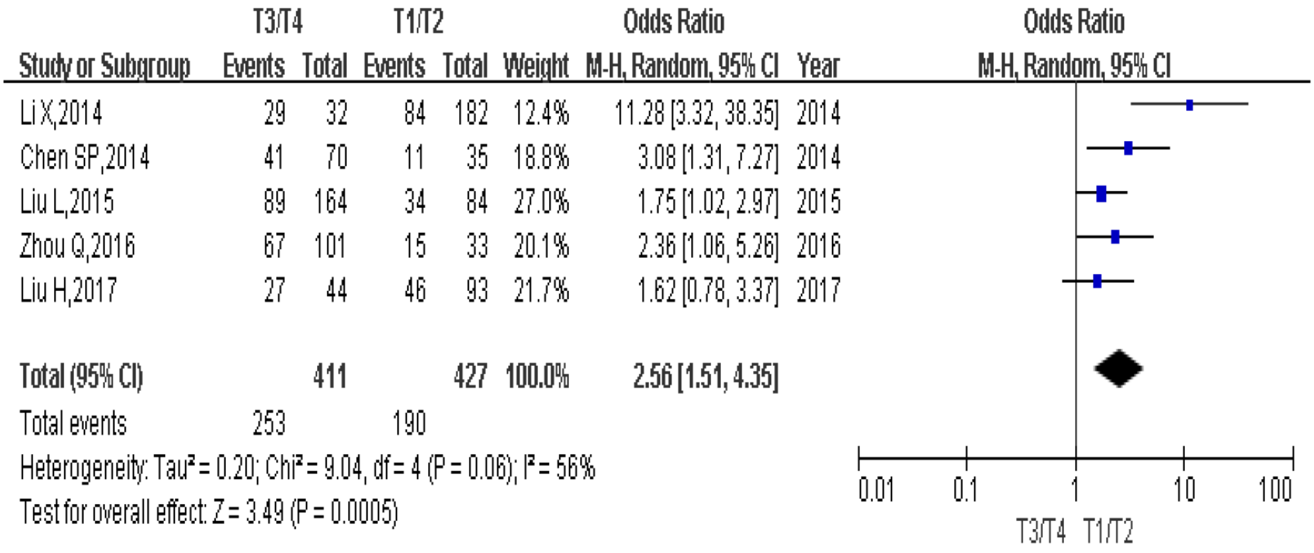

Supplementary Figure 7: The correlation between TBL1XR1 expression and tumor invasion.

Study or Subgroup	Poorly+Undifferentiated Events	Total	Well+Moderately Events	Total	Weight	Odds Ratio M-H, Random, 95% CI	Year
Wang J,2014	48	90	60	104	21.6%	0.84 [0.47, 1.48]	2014
Li X,2014	36	54	77	160	20.9%	2.16 [1.13, 4.11]	2014
Zhou Q,2016	49	76	33	58	20.5%	1.37 [0.68, 2.77]	2016
Kuang X,2016	34	39	18	68	17.0%	18.89 [6.40, 55.75]	2016
Liu H,2017	23	39	50	98	20.0%	1.38 [0.65, 2.92]	2017
**Total (95% CI)**		**298**		**488**	**100.0%**	**2.12 [0.93, 4.84]**	
Total events	190		238				

Heterogeneity: $Tau^2 = 0.74$; $Chi^2 = 26.30$, df = 4 (P < 0.0001); $I^2 = 85\%$
Test for overall effect: Z = 1.79 (P = 0.07)

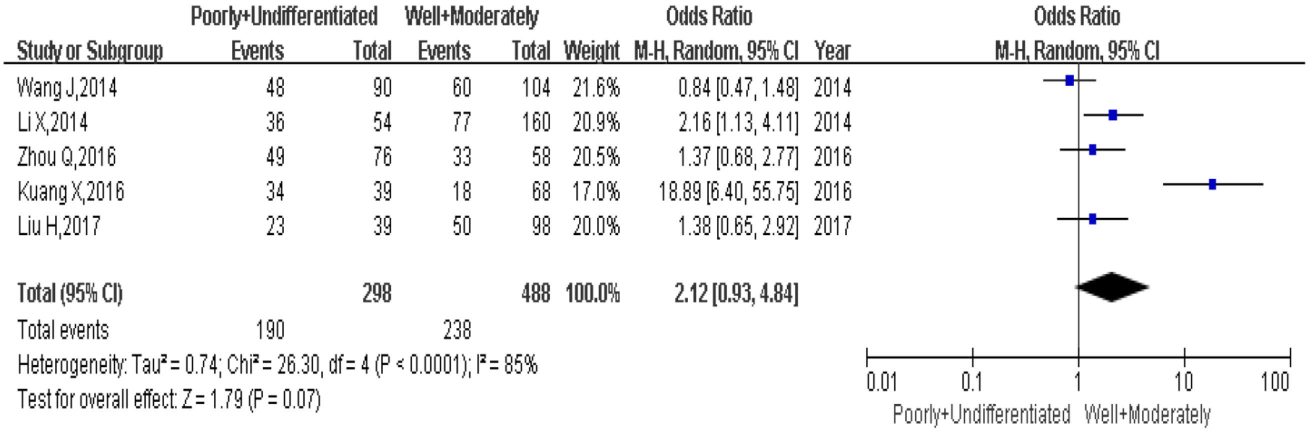

Supplementary Figure 8: The correlation between TBL1XR1 expression and tumor differentiation.

	High expression		Low expression			Odds Ratio	
Study or Subgroup	Events	Total	Events	Total	Weight	M-H, Random, 95% CI	Year
Li X,2014	81	108	66	106	18.1%	1.82 [1.01, 3.27]	2014
Chen SP,2014	44	52	35	53	14.4%	2.83 [1.10, 7.27]	2014
Liu L,2015	86	123	31	107	18.3%	5.70 [3.23, 10.06]	2015
Wu Y,2016	13	24	12	47	13.4%	3.45 [1.22, 9.72]	2016
Zhou Q,2016	48	82	29	52	16.9%	1.12 [0.56, 2.26]	2016
Liu F,2016	174	204	60	130	18.8%	6.77 [4.03, 11.37]	2016
**Total (95% CI)**		**593**		**495**	**100.0%**	**3.07 [1.66, 5.67]**	
Total events	446		233				

Heterogeneity: Tau² = 0.45; Chi² = 24.08, df = 5 (P = 0.0002); I² = 79%
Test for overall effect: Z = 3.59 (P = 0.0003)

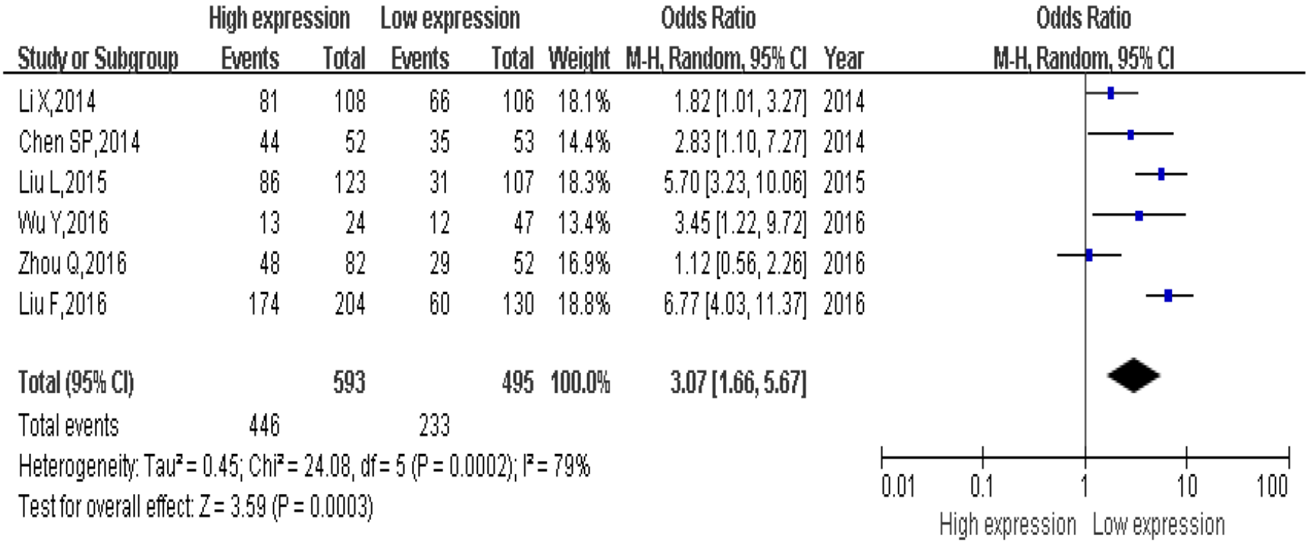

Supplementary Figure 9: The correlation between TBL1XR1 expression and lymph node metastasis.

	III/IV		I/II			Odds Ratio	
Study or Subgroup	Events	Total	Events	Total	Weight	M-H, Random, 95% CI	Year
Li X,2014	52	86	61	128	17.5%	1.68 [0.97, 2.92]	2014
Chen SP,2014	49	82	3	23	15.4%	9.90 [2.72, 36.01]	2014
Liu L,2015	84	112	39	118	17.5%	6.08 [3.42, 10.79]	2015
Zhou Q,2016	68	96	14	38	17.0%	4.16 [1.88, 9.20]	2016
Liu F,2016	204	217	5	117	16.2%	351.51 [122.17, 1011.38]	2016
Wu Y,2016	13	26	11	45	16.3%	3.09 [1.11, 8.63]	2016
**Total (95% CI)**		**619**		**469**	**100.0%**	**8.48 [2.25, 31.97]**	
Total events	470		133				

Heterogeneity: Tau² = 2.53; Chi² = 82.46, df = 5 (P < 0.00001); I² = 94%

Test for overall effect: Z = 3.16 (P = 0.002)

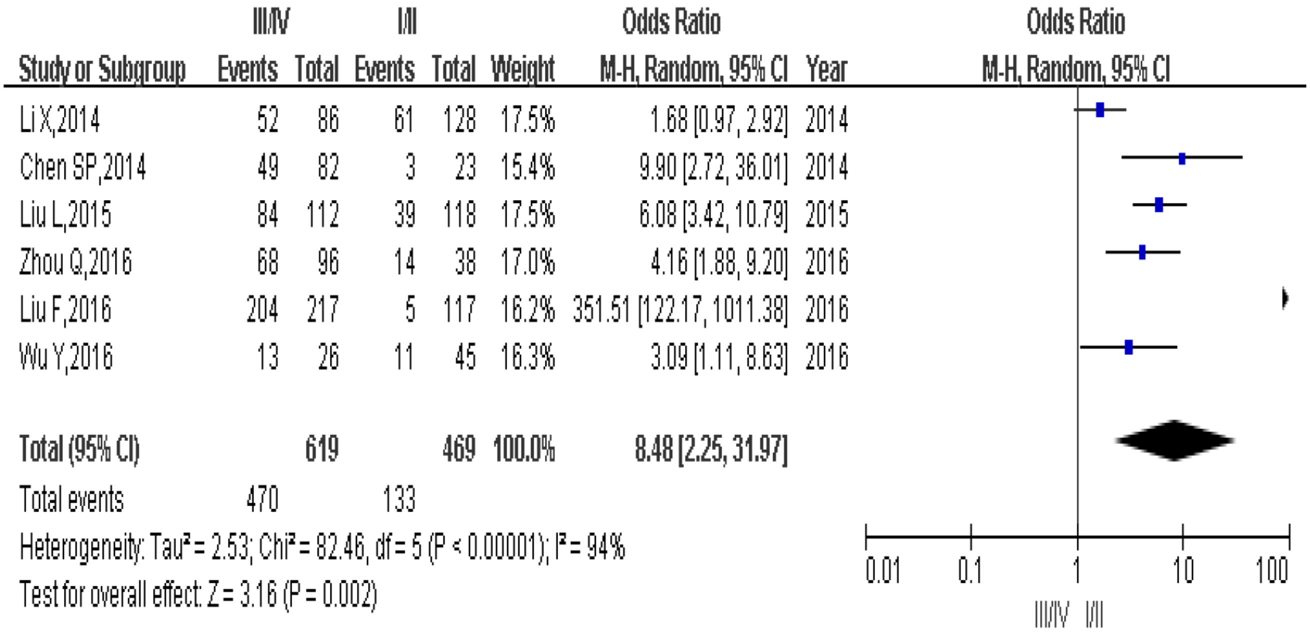

Supplementary Figure 10: The correlation between TBL1XR1 expression and TNM stage.
